# Why Must We Keep Discussing Strategic Purchasing and Managed Competition? Comment on "Measuring Active Purchasing in Healthcare: Analysing Reallocations of Funds Between Providers to Evaluate Purchasing Systems Performance in the Netherlands"

**DOI:** 10.34172/ijhpm.9248

**Published:** 2025-08-05

**Authors:** Scott L. Greer

**Affiliations:** ^1^School of Public Health, University of Michigan-Ann Arbor, Ann Arbor, MI, USA.; ^2^European Observatory on Health Systems and Policies, Brussels, Belgium.

**Keywords:** Strategic Purchasing, Managed Competition, Netherlands, England, Payment Systems

## Abstract

Stadhouders and colleagues’ new measure answers an important question: Do strategic purchasing and managed competition redirect healthcare resources, and, if so, when, how, and to what? Applying it to the Netherlands, they find that they do not. This commentary first examines logical problems in arguments for strategic purchasing and managed competition, and then briefly reviews other evidence of their very limited success from, in particular, the Netherlands and England. It then raises the question of why strategic purchasing and managed competition continue to be advocated despite the poor logic of the arguments behind them and substantial evidence that they do not work.

 Do strategic purchasing and managed competition redirect healthcare resources, and, if so, when, how, and to what? Stadhouders and colleagues^1^ construct a useful new measure to ask this important question. They use the measure to examine the impact of strategic purchasing in the Netherlands. The Netherlands is an important case for students of any form of market competition or purchasing in healthcare because it combines generally good governance, a broad political commitment to healthcare access, a relatively dense population with good transport connections that facilitate competition between facilities, and a large-scale reform that shifted its entire healthcare system to a private insurance model and which has been carefully evaluated by the authors and others^2,3^ as part of a global debate about the impact of competition and purchasing.^4^ If any country could make strategic purchasing and managed competition work, and generate the data needed to prove it, it would be the Netherlands.

 The results will disappoint advocates or health policy-makers who were hoping to use it to improve resource allocation: “Dutch managed competition and competitive purchaser reforms had no discernible effect on reallocations of funds between providers.” In what is probably the best implementation we will see for these policies, they did not do what markets are supposed to do best, namely reveal information and incentivize the reallocation resources to producers with a better relationship between price and quality.

 Why do these results matter so much? Because they touch on a major policy issue the world over. The global push for managed competition and strategic purchasing in healthcare has gone on for decades, and we have been seeing implementations of the ideas for at least forty years (dating back to Margaret Thatcher’s *Working for Patients*, which introduced the “internal market” and “purchaser-provider split” in the UK’s National Health Service [NHS] systems).^5^

 Even if, like much of neoliberalism, the intellectual elan is gone, the policy ideas are entrenched in systems that separate purchaser and provider, that encourage competition among providers or insurers, in assumptions of consultants, civil servants, and academics who simply accept these ideas and policies as reality, and in policy advocates of various sorts who suggest these ideas. (Though the United States Agency for International Development, a major proponent of strategic purchasing and competition in healthcare delivery in recent years, has been effectively destroyed.^6^ The ideas it was supporting will no longer be backed by a large US development aid budget).

 The results Stadhouders and colleagues found are also, unfortunately and unsurprising. The arguments that managed competition strategic purchasing would work were always built on unstable foundations. Stanford University economist Alain Enthoven is often credited with inventing the concepts of both strategic purchasing and managed competition, and so his definition of strategic purchasing is worth a look as a foundational text:

 “[A] purchasing strategy to obtain maximum value for consumers and employers, using rules for competition derived from microeconomic principles. A sponsor (either an employer, a governmental entity, or a purchasing cooperative), acting on behalf of a large group of subscribers, structures and adjusts the market to overcome attempts by insurers to avoid price competition. The sponsor establishes rules of equity, selects participating plans, manages the enrollment process, creates price-elastic demand, and manages risk selection.”^7^

 This definition is not well constructed by scientific standards. It incorporates the expected goals of a policy into its definition. It makes it easy for advocates to wiggle out of any disconfirming results – they can simply assert that the Dutch reforms were not sufficiently guided by microeconomic principles, the sponsor did not create properly price-elastic demand, policy-makers did not actually want to obtain maximum value for employers, or whatever. In the specific case of Stadhouders et al, they could argue that managed competition and strategic purchasing are two different things that cannot be simultaneously evaluated in one case.

 The list of required conditions for the argument to work is so impressive as to suggest that a system which attained them could probably make any model of decision-making work well. It is, in short, a rhetorical exercise for policy advocacy rather than a scientifically viable definition.^8^

 What it left out was how strategic purchasing was supposed to work in a real world where not only the extensive list of preconditions was often missing but the problems that the concept faced mattered. Strategic purchasing and managed competition, we have argued,^9^ always faced the problem that purchasers of healthcare lacked information relative to the doctors and patients, lacked political popularity relative to doctors, patients and hospitals and power relative to the politicians who support what is popular, lacked capital relative to states (since capital expenditure was outside the US not effectively incorporated into their payment rates), and lacked alternative suppliers in largely public systems with major barriers to entry and exit. To the extent that they could address these asymmetries the remedies were extremely costly and might also be unpopular, as with expensive health information technology programs and efforts to ration treatments or avoid expensive patients. It should be no surprise if the result is, as in the Netherlands, a costly and elaborate superstructure that requires extensive ongoing regulation and provides no clear advantages relative to alternative organizational forms. Nor should it be a surprise that in the COVID-19 pandemics governments swept payers aside from their central roles in purchasing, since little about their operations was suited to crisis conditions.^10^ Further, its focus on dis-integration in the name of competition and accountability through contracting also makes it harder to join up policy, which means that adoption of managed competition and strategic purchasing can interfere with broader goals such as addressing health inequality, investing in prevention, or coping with difficult problems such as mental health provision (in turn, of course, there are now efforts in systems that adopted managed competition and strategic purchasing to use payment reforms to address these issues).

 In the English NHS, which has the longest experience of life under managed competition, it tends to be a luxury. Competition is swiftly replaced by local networks and cooperation whenever there is austerity.^11,12^ Purchasing, when examined in practice, turns out to be a largely consensual and political rather than competitive or reallocating process.^13,14^ If we are looking at key cases, the Dutch might have been the likeliest implementation to succeed, but the English have tried for the longest, so if their results are the same then the rest of us should take note.

 One could read Stadhouders et al and other articles as the latest in a long series of highly competent evaluations of best-case implementations of a basic idea that does not do what it is supposed to do. In that case, adding more goals such as value-based purchasing, health technology assessment, care integration, skillmix changes or better transitions to long term care raises two concerns. One is conceptual: if we pile still more goals atop Enthoven’s heap, how can we possibly evaluate the basic idea? The other is practical: while an existing strategic purchasing or managed competition system might lead to, for example, value-based payment as a useful tool, the usefulness of that tool in the context of those systems might not be enough to support a case for adopting such systems.

 So why, then, has a poorly specified concept that has fared badly in policy evaluations, including this important one, new persist? Like user fees, it seems to be a “zombie idea” that simply cannot be killed by experience or research.

 Part of its resilience might lie in its poor specification. The essentially rhetorical advocacy approach that Enthoven created might help to explain its persistence despite evidence of disappointment. Who would oppose such wholesome-sounding concepts as managed competition and strategic purchasing (in favor of what, disregulated monopoly and haphazard spending? As Marmor pointed out, the names are tendentious).^8^ Policy failures elsewhere, if they are noticed at all, can be explained away by context – failure to fulfill all the necessary conditions. This is why the Dutch case matters so much. It was a thoroughgoing and very public reform that did most of what advocates wanted and has received high quality evaluations. Another reason for the persistence of strategic purchasing and managed competition might lie in their broad congruence with other elements of what we might call neoliberalism – a focus on markets, on focused accountability for organizations, on “getting the prices right,” a suspicion of direct state provision.^15^

 Interest group politics are also a potential factor; providers and private insurers might both feel that they would benefit from the rent-seeking opportunities created by additional complexity that managed competition and strategic purchasing can create in a public system. Consulting firms have global experience that they can claim enables them to help design and implement complex market systems. More long-term players might be interested in creating opportunities for new private actors to enter lucrative parts of the healthcare system by setting up markets in which they can participate. Ideological advocates of markets can blend with ideological, or wholly self interested, advocacy of privatization in systems fragmented by managed competition and a focus on purchasing. Private equity firms are increasing good at identifying, entering, and even creating profitable niches in fragmented healthcare systems.^16^

 The logic of policy design also might help to explain strategic purchasing’s attractiveness. Healthcare systems constantly purchase, whether by paying workers or buying medicines. Fashioning care into procedures that can be bought and sold, eg, by creating diagnosis related groups, means that instead of policy-makers buying inputs such as hospital days, they can try to buy what they actually want, which is healthcare. The data created by internal markets can be useful, if biased towards payments. Contracts can provide a focus for planning and strategy as well as a measure of accountability if budgets or care planning go wrong.

 Finally, it is possible that strategic purchasing or managed competition can simply be umbrella terms for efforts to run systems better. Rather than thinking of either as a policy idea that can be adopted, evaluated, and diffused, this perspective would see it as a bundle of ideas to make existing managed competition systems less cumbersome and more likely to produce clarity and accountability and ideally quality, access, responsiveness, or efficiency.

 The world is full of putative strategic purchasing and managed competition systems, and regardless of whether the policy design that created them was good, they still need managing and can still be made to function better or worse.^4^ Policy instruments such as payment system reforms, competition (antitrust) enforcement, and health technology assessment can work well within such actually existing systems to promote quality, efficiency, and cost containment. Policy-makers should not bother to make them fulfill advocates’ rhetorical promise but instead try to make the best of them by using the data and accountability they create without expecting any market miracles to result.

## Ethical issues

 Not applicable.

## Conflicts of interest

 Author declares that he has no conflicts of interest.
